# Longitudinal Impact of Depressive Symptoms and Peer Tobacco Use on the Number of Tobacco Products Used by Young Adults

**DOI:** 10.3390/ijerph182111077

**Published:** 2021-10-21

**Authors:** Caroline North, C. Nathan Marti, Alexandra Loukas

**Affiliations:** Department of Kinesiology & Health Education, University of Texas at Austin, Austin, TX 78712, USA; cen843@utexas.edu (C.N.); nate.marti@utexas.edu (C.N.M.)

**Keywords:** tobacco products, depression, peers, young adults, Texas, longitudinal

## Abstract

We examined the role of depressive symptoms in the longitudinal trajectory of the number of tobacco products used across young adulthood, ages 18–30 years, and whether peer tobacco use exacerbated the effects of the depressive symptoms. Participants were 4534 initially 18–25-year-old young adults in the Marketing and Promotions Across Colleges in Texas project (Project M-PACT), which collected data across a 4.5-year period from 2014 to 2019. Growth curve modeling within an accelerated design was used to test study hypotheses. Elevated depressive symptoms were associated with a greater number of tobacco products used concurrently and at least six months later. The number of tobacco-using peers moderated the association between depressive symptoms and the number of tobacco products trajectory. Young adults with elevated depressive symptoms used a greater number of tobacco products but only when they had a greater number of tobacco-using peers. Findings indicate that not all young adults with depressive symptoms use tobacco. Having a greater number of tobacco-using peers may facilitate a context that both models and encourages tobacco use. Therefore, tobacco prevention programs should aim to include peer components, especially for young adults.

## 1. Introduction

Young adults (aged 18–25 years old) have the highest prevalence of both single tobacco and nicotine (hereafter referred to as tobacco) product use [1], as well as concurrent use of two or more products (i.e., poly-tobacco use; [2]). Data from the Population Assessment of Tobacco and Health (PATH) study indicate that in 2014, 21.7% of young adults were current poly-tobacco users [3]. Young adulthood is also a time when there are multiple and frequent changes in tobacco use including initiation, escalation of use, transitions to consistent use, declines in use, and quitting [4,5,6,7]. Although limited, recent research indicates that tobacco use declines as young adults age [8,9,10]. For example, desistance (i.e., quitting) of electronic nicotine delivery systems (ENDS) across a two-year period among young adults is common [10] and longitudinal research indicates poly-tobacco use declines from ages 18 to 28 [9]. According to the maturing out hypothesis [11], desistance and/or declines in substance use, including tobacco, may be likely among young adults as they age due to substance use not being compatible with adult roles, such as full-time employment or marriage. Yet, relatively few contemporary studies examine factors associated with longitudinal trajectories of poly-tobacco use across young adulthood. The purpose of the present study was to examine the roles of depressive symptoms and peer tobacco use in the longitudinal trajectory of the number of tobacco products used across young adulthood.

Not all young adults are equally vulnerable to tobacco initiation and progression, there are disparities in use and cross-sectional evidence indicates the co-occurrence of depression and tobacco use is common [12,13]. Depression, one of the most common mental health disorders in the United States [14,15,16], peaks during young adulthood [15]. Individuals with depression, including those with symptoms of depression, use tobacco products, either alone or concurrently, at high rates [12,13,17,18,19]. Researchers have hypothesized that individuals who suffer from depression and/or experience high levels of depressive symptoms may use tobacco as a coping strategy to self-regulate mood [18].

Numerous studies have identified the co-occurring association between depressive symptoms and tobacco use. For example, cross-sectional and retrospective research indicates that experiencing elevated depressive symptoms in adulthood is associated with a number of smoking outcomes including smoking a greater number of cigarettes, lower odds of cigarette cessation, and higher odds of relapse [12,13,20,21]. Depressive symptoms are also concurrently linked to greater use of alternative tobacco products, including hookah and ENDS [22,23,24]. One longitudinal study reported depressive symptoms predicted ENDS use up to six months and one year later among young adults [25]. Similarly, adults experiencing elevated depressive symptoms are at greater odds of dual or poly-tobacco use [17,26]. However, little is known regarding the longitudinal associations between depressive symptoms and tobacco product use, including the number of products used during young adulthood [18], when not only are the prevalence of tobacco use and past-year depression highest [1,27] but when there are also frequent changes in tobacco use patterns [2,5,6,7,10].

Although depressive symptoms are associated with poly-tobacco use, evidence for the strength of the reported associations is inconclusive [18]; that is, not everyone who experiences depressive symptoms also uses tobacco products. Thus, there may be contextual influences that determine for whom and under what conditions depressive symptoms are associated with tobacco use. One such contextual influence is peer (i.e., friend) tobacco use. Peer use continues to be an important determinant of tobacco product use across young adulthood [28,29,30,31]. Yet, the role of peer tobacco use in the associations between young adults’ depressive symptoms and trajectories of tobacco use across young adulthood is not known. Since peer tobacco use is positively associated with young adults’ tobacco use [30], peer use may play a moderating role in this association, exacerbating the impact of depressive symptoms on young adults’ tobacco use trajectories. In particular, young adults with elevated depressive symptoms may be particularly likely to rely on peers for emotional support [32]. Therefore, peer tobacco use may provide a context that models and encourages tobacco use [33], and, in turn, exacerbates depressive symptoms.

### Current Study

Growth curve modeling was used to examine the roles of depressive symptoms and peer tobacco use in the longitudinal trajectory of the number of tobacco products used across young adulthood, ages 18–30 years. Prior research illustrates that the number of tobacco products young adults use declines as they increase in age [9]. We examined (1) the role of depressive symptoms in the declining tobacco use trajectory across ages 18–30 and (2) if peer tobacco use served as a moderating factor in the association between depressive symptoms and the number of tobacco products trajectory. It was hypothesized that (1) elevated levels of depressive symptoms would be associated with a greater number of tobacco products used across ages 18–30 and (2) having a greater number of tobacco-using peers would significantly exacerbate any associations observed in Hypothesis 1, such that the aforementioned associations would be stronger among young adults with a greater number of tobacco-using peers compared with those with fewer tobacco-using peers.

## 2. Materials and Methods

### 2.1. Participants

Participants were 4534 young adults participating in the rapid-response longitudinal Marketing and Promotions Across Colleges in Texas project (Project M-PACT). Project M-PACT recruited 5482 college students who were 18–29 years old at entry into the project in fall 2014/spring 2015 and followed for 4.5 years until spring 2019. Students were recruited from 24 2-and 4-year colleges across 5 counties surrounding four large Texas metropolitan areas (San Antonio, Houston, Dallas/Fort Worth, and Austin). Over 13,000 students (*N* = 13,714) were eligible to participate, and roughly 40% (*n* = 5482) provided consent and completed the survey. 

For the purposes of the present study, only data from students who were 18–25 years old, an age range typically considered young adulthood, at Wave 1 were included in this study (*n* = 5221). Participants were excluded from the current study sample if they did not provide complete data on the socio-demographic covariates or other key measures. Specifically, of the 5221 participants who were 18–25 years old at Wave 1, 687 cases were excluded due to missing socio-demographic data and incomplete data on the outcome and all focal variables, resulting in a sample size of 4534 for the present study. At Wave 1, the 4534 participants were 18–25 years old (*M* = 20.6; *SD* = 1.81), 64.2% were female; 93.7% attended a 4-year college compared with a 2-year college; 35.2% were non-Hispanic white, 31.0% Hispanic/Latino, 18.3% Asian, 7.9% African American/Black, and 7.6% reported two or more races/ethnicities, or another race/ethnicity.

### 2.2. Procedures

All participants were recruited in fall 2014/spring 2015 via email and were asked to provide consent online before enrolling in the web-based study. Follow-up web surveys were administered every six months until spring 2018, and then a yearly survey was administered in spring 2019. In fall 2017, an abbreviated survey was administered that was not used for this study because it included only limited current tobacco use data. Thus, this study used data from eight waves (with six months between the first six waves and then one year between the remaining two waves) spanning a 4.5-year period from 2014 to 2019. Participants received an incentive at each completed study wave: USD 10 e-gift card at Waves 1 and 2, a USD 20 e-gift card at all other full study waves, and a USD 5 e-gift card for the abbreviated survey. Retention rates for the 5482 participants at waves 2 through 8 ranged from 72.6% (Wave 8) to 84.5% (Wave 2). All study procedures were Institutional Review Board approved (IRB; 2013-06-0034), and additional information regarding Project M-PACT procedures can be located elsewhere [34].

### 2.3. Measures

Socio-demographic Covariates. Sex (coded 1 if male, 0 otherwise), age (centered at 18), college type (2 years versus 4 years; coded 1 if the participant attended a 4-year institution at Wave 1, 0 otherwise), and race/ethnicity, all assessed at Wave 1, were included in the models as covariates. The race/ethnicity variable was created by combining two items, one that assessed Hispanic/Latino/a ethnicity and the other that assessed race. Participants that identified as Hispanic or Latino/a were coded as Hispanic and all remaining participants were coded based on the race they selected (“*White*”, “*Black or African American*”, “*Asian*”, “*American Indian or Alaska Native*”, “*Native Hawaiian or Pacific Islander*” and “*Other*”). All participants endorsing more than one of the aforementioned races were coded as “other”. Race/ethnicity was dummy coded to indicate Hispanic/Latino, African American/Black, Asian American, and other compared with non-Hispanic White, which served as the reference group.

#### 2.3.1. Dependent Variable

*Tobacco and Nicotine Product Use*. Current tobacco product use was assessed at all waves using items adapted from the Youth Tobacco Survey [35] and the PATH study [36]. Past 30-day use of five types of tobacco products was assessed at each wave, including cigarettes, smokeless/snus tobacco, large cigars/cigarillos/little cigars, hookah, and ENDS. Current use of cigarettes and smokeless tobacco was queried with “*During the past 30 days, on how many days did you smoke/use__?*” Current use of large cigars/cigarillos/little cigars and hookah was queried with “*During the past 30 days, how many days did you smoke ____ as intended (i.e., with tobacco)?*” Current ENDS use was queried with “*During the past 30 days, have you used any ENDS product (i.e., an e-cigarette, vape pen, or e-hookah), even one or two puffs, as intended (i.e., with nicotine cartridges and/or e-liquid/e-juice)?*” Beginning in spring 2018, JUUL/pod-vapes were also included as example products in the ENDS products use item. Each type of five tobacco products was assigned a score of 0 (used on 0 days within the past 30) or 1 (used on at least 1 day within the past 30). Scores were summed to create an index ranging from 0 to 5, reflecting the number of tobacco products used in the past 30 days at each wave.

#### 2.3.2. Independent Variable

*Depressive Symptoms*. The 10-item short-form of the Center for Epidemiological Studies-Depression-10 Scale (CES-D-10) was used to assess depressive symptoms [37] at all eight waves. This measure assessed the frequency of past week symptoms of depression including somatic complaints, depressed affect, and positive affect. Each item was scored on a scale ranging from 0 “*Rarely (<1 Day)*” to 3 “*Most of the time (5–7 Days)*”. Two items, querying about feeling happy or hopeful, were reverse coded. All 10 items were summed and higher scores reflected greater levels of depressive symptoms. The CES-D-10 is both a reliable and valid measure of depressive symptoms among adults [38]. Internal consistency reliability of the 10-item scale ranged from Cronbach’s α = 0.80 to Cronbach’s α = 0.85 at each of the eight waves, from which data were drawn for the current study. The variable was mean-centered to improve the interpretation of model parameters. 

#### 2.3.3. Moderating Variable

*Peer Use*. Peer tobacco and nicotine product use was assessed at all eight waves using items adapted from the PATH Study [37]. Peer use of five products, cigarettes, smokeless/snus tobacco, large cigars/cigarillos/little cigars, hookah, and ENDS was assessed with the item “*How many of your close friends smoke/use ____?*” Response options ranged from 1 (“*None*”) to 5 (“*All*”). A composite score was created that represented the mean across the five tobacco product scales and thus ranged from one to five. The variable was mean-centered to improve the interpretation of model parameters.

### 2.4. Data Analysis

We evaluated both concurrent and lagged measures of depressive symptoms and of peer tobacco use on the trajectory of the number of tobacco products. Concurrent measures are those that were assessed within the same wave as the outcome, whereas lagged measures were assessed in the wave prior to the outcome. As lagged effects require data assessed at the wave prior to the outcome variable, the first instance of the outcome was at Wave 2 (spring, 2015). Although the depressive symptoms and peer tobacco use independent variables were assessed at all eight waves, beginning at Wave 1, the trajectory of the number of tobacco products included only data from Wave 2 and beyond. 

Growth curve modeling within an accelerated longitudinal design was used to determine the roles of depressive symptoms and peer tobacco use in the longitudinal trajectory of the number of tobacco products used across young adulthood, ages 18–30 years. The accelerated design used participant age to assess change. Participants contributed up to four years of outcome data (i.e., the number of years between spring 2015 and spring 2019) on the basis of their exact age at each study wave. Therefore, the full trajectory represents the number of products used from ages 18–30, which is the entire age range of the sample, estimated from the overlapping age ranges from individual participants within each wave. To account for the nonindependence of observations due to the nested structure of the data, whereby individual waves were nested within participants, and participants were nested within the college they attended, all models included random intercepts for the participant and the college that they attended. Analyses were conducted in R-Studio using the lmer function from the lme4 package [38].

Model building occurred in two stages. The goal of the first stage of model building was to establish the rate and shape of the trajectory of the number of tobacco products used across the sample’s age range. Following recommendations from Singer and Willett [39], unconditional growth curve models (i.e., models with only temporal variables) were fit to establish the trajectory of the number of tobacco products used across young adulthood, from ages 18–30 years. First, separate unconditional growth models were fit in which age was treated as linear, quadratic, and log transformed. The unconditional growth models were compared using the Bayesian Information Criterion (BIC), which accounts for both model complexity and sample size in determining the best-fitting model [40]. The best-fitting unconditional growth curve model (i.e., lowest BIC) was the linear model, which exhibited a negative trend across increasing age (*t* (25,269) = −8.80, *p* < 0.001), indicating that the number of tobacco products used declined as participants increased in age. In the second stage of model building, conditional growth models were fit by adding the three time-invariant socio-demographic variables (participant sex, 2- versus 4-year college, and race/ethnicity) and the time-varying variables, depressive symptoms (concurrent and lagged), and peer tobacco use (concurrent and lagged), to the model.

#### 2.4.1. Depressive Symptoms Model 

After establishing linear age as the unconditional growth model and adding socio-demographic covariates, the two-way interactions between age × concurrent depressive symptoms and age × lagged depressive symptoms were examined to test Hypothesis 1 and determine if depressive symptoms predicted change in the trajectory of the number of tobacco products across ages 18–30. However, examination of the BIC indicated that the model fit was not improved when including the two interactions, compared with models with just main/independent effects of both concurrent and lagged depressive symptoms. Thus, depressive symptoms did not amplify or attenuate the rate of change in the number of tobacco products used as participants increased in age. Therefore, the two-way interaction between age and depressive symptoms (both concurrent and lagged) was not included in subsequent models. 

#### 2.4.2. Peer Tobacco Use Moderation Model

Next, in order to test Hypothesis 2 and examine whether peer tobacco use served as a moderating/exacerbating factor in the association between depressive symptoms and the trajectory of the number of tobacco products, concurrent and lagged peer tobacco use variables were included in the model as both main/independent effects and in two-way interactions with depressive symptoms (i.e., concurrent peer tobacco use × concurrent depressive symptoms, and lagged peer tobacco use × lagged depressive symptoms). The R emtrends function [41] was used to examine significant interaction effects by using model-based estimates of the relationship between depressive symptoms and the number of products used (i.e., the depressive symptoms simple slope) at one standard deviation (SD) above and below the mean of peer tobacco use.

#### 2.4.3. Attrition Analyses

Separate mixed logistic regression models were fit for each of the four Wave 1 socio-demographic covariates (sex, college type (2- versus 4-year), and race/ethnicity) and time-varying participant age (centered at age 18) to determine if missing observations at each time point for participants included in the present study (*N* = 4534) varied from participants in the larger cohort that would have otherwise been eligible but were not included due to missing data (*n* = 684). Data were more likely to be missing only for participants that were older (odds ratio (OR) = 1.25, 95% confidence interval (CI) = 1.22, 1.28) and male (OR = 1.29, 95% CI = 1.08, 1.55), and less likely to be missing for Asian American participants versus non-Hispanic, white (OR = 0.57, 95% CI = 0.44, 0.74). However, the effect sizes were small, and there were no significant differences on any other socio-demographic covariates.

## 3. Results

Table 1 contains the mean and standard deviations for the independent, moderator, and dependent variables at each study wave. The mean number of tobacco products used was highest in spring, 2015 (M = 0.53) and lowest in spring, 2019 (M = 0.38). Peer tobacco use was highest in the fall, 2014 (M = 1.85) and lowest 2.5 years later in spring 2017 (M = 1.62), whereas depressive symptoms were lowest in fall 2015 (M = 6.92) and highest six months later in fall 2015 (M = 8.42). Examination of the means at each study wave indicated that the number of tobacco products used by participants and the number of tobacco-using peers appeared to decrease across the 4.5-year study period, but there was no clear pattern for depressive symptoms. 

### 3.1. Depressive Symptoms Model

The role of depressive symptoms in the tobacco use trajectory across young adulthood was examined by regressing the declining trajectory of the number of tobacco products on the main effects of concurrent and lagged depressive symptoms (Hypothesis 1). Consistent with the unconditional growth model, results indicated that age was negatively associated with the number of products used trajectory, indicating that the number of tobacco products used declined as young adults increased in age. Results also showed that there was a significant positive effect for concurrent depressive symptoms and lagged depressive symptoms (see Table 2). Young adults with greater depressive symptoms reported using a greater number of tobacco products concurrently and subsequently, at least six months later. Examination of the remaining socio-demographic covariates indicated that only sex and Asian American race/ethnicity predicted the trajectory of the number of tobacco products. Participants who were Asian American (compared with non-Hispanic white participants) used fewer tobacco products across the range of the study, while males used more tobacco products than females.

### 3.2. Peer Tobacco Use Moderation Model

**Figure 1:**
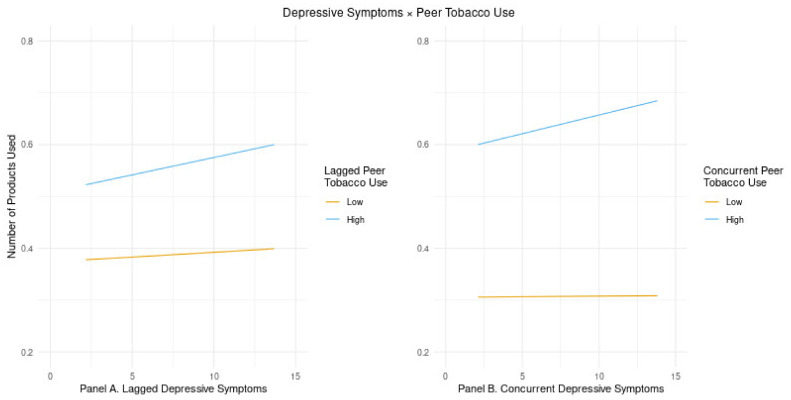
Examining significant two-way interactions between depressive symptoms and peer tobacco use across the longitudinal trajectory of the number of tobacco products used in the past 30 days (*N* = 4534). Lines represent the model-estimated relationship between the number of tobacco products used and depressive symptoms graphed separately at low and high values of peer tobacco use where the low and high values are the peer tobacco use at one standard deviation below and above the mean, respectively.

Two-way interactions between depressive symptoms and peer tobacco use were added to the depressive symptoms model to examine whether having a greater number of tobacco-using peers would significantly moderate any associations between depressive symptoms and the trajectory of tobacco products across ages 18–30 (Hypothesis 2). Similar to the main effects model, concurrent depressive symptoms and lagged depressive symptoms remained significant. In addition, concurrent peer tobacco use and lagged peer tobacco use were positive and significant (see Table 2). The concurrent two-way interaction between depressive symptoms and peer tobacco product use was also significant, as was the lagged interaction (see Table 2). Both interactions indicate that peer tobacco use significantly exacerbated the association between depressive symptoms and the number of tobacco products used, such that young adults experiencing elevated depressive symptoms used a greater number of tobacco products but only when they had a greater number of tobacco-using peers. The interaction effects are displayed in Figure 1, in which the relationship between the number of products used and depressive symptoms (i.e., the slope) estimated at 1 SD above and 1 SD below the peer tobacco use mean are displayed. Probing the significant lagged depressive symptoms × lagged peer tobacco use interaction indicated that the association between the number of tobacco products used and lagged depressive symptoms (i.e., the lagged depression simple slope) was not significant at 1 SD below the lagged peer tobacco use mean (95% CI = (<−0.001, 0.004)) but was at 1 SD above the mean (95% CI = (0.005, 0.009)) (see Figure 1, Panel A). Probing the significant concurrent depressive symptoms × concurrent peer tobacco use interaction indicated that the association between the number of tobacco products used and concurrent depressive symptoms (i.e., the concurrent depression simple slope) was not significant at 1 SD below the concurrent peer tobacco use mean (95% CI = (−0.002, 0.002)) but was significant at 1 SD above the mean (95% CI = (0.005, 0.009)) (see Figure 1, Panel B). 

## 4. Discussion

Young adulthood is a time when there are multiple and frequent changes in tobacco use [4,5,6,7]. Limited research indicates that young adults use fewer tobacco products as they increase in age [8,9,10]. However, relatively few studies examine longitudinal trajectories of tobacco product use across young adulthood, including the potential factors associated with the tobacco use trajectories. Findings confirm existing research indicating that the number of tobacco products declined as young adults increased in age. Study findings also extend existing research by showing that young adults experiencing elevated depressive symptoms used a greater number of tobacco products but only when they had a greater number of tobacco-using peers. Results from the current study provide unique data that can be used to identify subgroups of young adults with depressive symptoms who are most vulnerable to single and poly-tobacco use.

Findings from the current study are consistent with prior research indicating that depressive symptoms are associated with a greater likelihood of tobacco product use [17,26]. Although depressive symptoms did not amplify or attenuate the rate of decline in the number of tobacco products used as participants increased in age, current and lagged depressive symptoms were both associated with the number of tobacco products used by young adults concurrently and at least six months later. Although the present study did not assess how or why depressive symptoms elevated the risk for tobacco use, researchers have suggested that young adults with elevated depressive symptoms may engage in tobacco product use in an attempt to self-regulate or alleviate negative affect and emotional distress [18,26]. These findings are concerning given recent evidence that an increasing number of young adults suffer from depression [42]. Subsequent research should therefore aim to assess reasons for tobacco use among young adults with elevated depressive symptoms to further understand the association between depressive symptoms and tobacco use. 

Consistent with expectations, peer tobacco use moderated the associations between depressive symptoms and the number of tobacco products used. In fact, depressive symptoms were associated with a greater number of tobacco products used but only among young adults with a greater number of tobacco-using peers. These findings are consistent with evidence indicating that peers continue to play an important role in young adults’ tobacco use behaviors [30]. Those experiencing depressive symptoms may rely on peers for emotional support [32]. However, peer tobacco use may also provide a unique social and environmental context that both encourages and models tobacco use [34]. For example, young adults are more likely to engage in risk-taking behaviors (e.g., tobacco use) when in the presence of peers [43], and social cognitive theory suggests that young adults may be more likely to follow the influence of peers due to the increase in social interaction alone. Although prior research has primarily focused on the influence of peer networks on adolescent tobacco use, the influence of peers on tobacco product use in young adulthood is likely stronger due to young adults often having larger social networks, increased autonomy, and easier access to products [44]. Therefore, future research should further assess the influence of peer networks on tobacco use in young adulthood. 

The current study has a number of strengths, foremost of which is the longitudinal design and the large and diverse sample of young adults. However, there are a few limitations. First, participants were recruited from colleges in Texas, and therefore cannot be generalized to all young adults. Future research should assess the role of depressive symptoms and peer tobacco use in the tobacco use trajectory among a more representative sample of young adults. In addition, the current study did not assess the clinical diagnosis of depression. However, elevated depressive symptoms are a strong predictor of major depression [45], and compared with clinical depression, elevated depressive symptoms are also more prevalent within the general population [46]. The CES-D-10, our measure of depressive symptoms, is also a valid and economical way of measuring depressive symptoms on a continuum amongst large community samples [47]. Further, we examined longitudinal changes in the number of tobacco products used across young adulthood, and assessments were based on past 30-day use; therefore, we cannot determine who, if, or when participants became regular users of these tobacco products. Future research should continue to follow young adults as they transition into adulthood in order to longitudinally capture varying levels of tobacco use behaviors. Finally, although prior research shows that the association between depression and tobacco use is stronger for females than males [13], the current study did not assess differences in the depressive symptoms and number of tobacco products used association by sex. Thus, future research should aim to examine whether participant sex moderates the associations tested within the present study.

## 5. Conclusions

Despite these limitations, the present study’s findings are important and unique because they illustrate that in young adulthood, depressive symptoms play a significant role in the number of tobacco products used across time but only among those with a greater number of tobacco-using peers. These results can be used in the identification of subgroups of young adults most vulnerable to both single and poly-tobacco use, such as those with elevated depressive symptoms and greater tobacco-using peers. The current study’s results also have tobacco use intervention development implications. Specifically, peer network-based interventions among young adults that are socially oriented and/or experience high depressive symptoms may prove useful. For example, a social network text-based intervention, developed by Mason et al. [48], increased college student willingness to change their substance use behaviors by providing students with feedback on the substance use behaviors of their peers. College students were asked to reflect on their desired goals in college, and the ways they might adjust their social network (e.g., remove substance-using peers from social network) to meet their behavioral goals. Tobacco control efforts may also consider partnering with young adults to aid in the dissemination of anti-tobacco campaign materials. The utilization of young adult peers to disseminate anti-tobacco sentiments in order to de-normalize tobacco use on college campuses has been shown to be effective [49].

## Figures and Tables

**Table 1:** Mean and standard deviation for primary independent and dependent variables (*N* = 4534) by study wave, 2014–2019.

Variable Name	Study Waves							
	Fall, 2014	Spring, 2015	Fall, 2015	Spring, 2016	Fall, 2016	Spring, 2017	Spring, 2018	Spring, 2019
Number of tobacco products used	*	0.53 (0.94)	0.47 (0.88)	0.45 (0.81)	0.41 (0.81)	0.41 (0.80)	0.41 (0.80)	0.38 (0.77)
Peer tobacco use	1.85 (0.65)	1.79 (0.65)	1.73 (0.58)	1.69 (0.57)	1.66 (0.57)	1.62 (0.56)	1.65 (0.57)	1.63 (0.55)
Depressive symptoms	7.82 (5.38)	6.92 (5.17)	8.42 (5.78)	8.37 (5.92)	8.12 (6.03)	8.09 (6.04)	7.96 (5.94)	8.07 (5.90)

Note. Mean (SD). * As lagged effects require data assessed at the wave prior to the outcome variable (i.e., number of tobacco products used), the first instance of the outcome was in spring 2015.

**Table 2:** Multilevel growth curve model examining the longitudinal associations between depressive symptoms and peer tobacco product use and changes in number of tobacco products used (*N* = 4534).

	Depressive Symptoms Model					Peer Tobacco Use Moderation Model				
Parameter	Coefficient	*SE*	*df*	*t*	*p*	Coefficient	*SE*	*df*	*t*	*p*
Intercept	0.49	0.05	121	9.03	<0.001	0.40	0.05	175	8.78	<0.001
Age	−0.02	0.00	25,220	−9.27	<0.001	−0.01	0.00	24,091	−3.50	<0.001
Depressive symptoms	0.01	0.00	25,761	6.86	<0.001	0.00	0.00	25,068	4.50	<0.001
Depressive symptoms (lagged)	0.01	0.00	24,709	6.75	<0.001	0.00	0.00	25,024	5.12	<0.001
Peer tobacco use						0.29	0.01	25,097	33.19	<0.001
Peer tobacco use (lagged)						0.14	0.01	25,128	17.08	<0.001
Male sex	0.27	0.02	4360	12.53	<0.001	0.23	0.02	4141	12.04	<0.001
Hispanic/Latino	−0.03	0.03	3888	−0.97	0.332	−0.01	0.02	3470	−0.49	0.621
African/Black	−0.05	0.04	3383	−1.17	0.242	0.00	0.04	2962	−0.06	0.950
Asian	−0.10	0.03	4234	−3.38	0.001	−0.06	0.03	3937	−2.10	0.036
Other race/ethnicity	0.05	0.04	4364	1.16	0.246	0.04	0.04	4121	1.09	0.275
4-year institution	0.03	0.06	46	0.45	0.657	0.03	0.05	58	0.67	0.508
Depressive symptoms × Peer tobacco use						0.01	0.00	24,233	5.11	<0.001
Dep symptoms (lagged) × Peer Tobacco use (lagged)						0.00	0.00	24,035	3.48	<0.001

## Data Availability

Readers are encouraged to email alexandra.loukas@austin.utexas.edu for more information on the data for this study.
